# Peripheral blood regulatory T cells and disease activity, quality of life, and outcomes in children with juvenile idiopathic arthritis

**DOI:** 10.1186/s12969-024-01006-x

**Published:** 2024-08-01

**Authors:** Neus Quilis, Pablo Mesa-del-Castillo Bermejo, Paula Boix, Oriol Juanola, Pilar Bernabeu, Rubén Francés, Mariano Andrés

**Affiliations:** 1 Rheumatology Unit, Hospital Universitario Vinalopó, Servicio de Reumatología, C/ Tonico Sansano Mora 14. ZIP: 03293, Elche, Alicante, Spain; 2 Paediatric Rheumatology Department, Virgen de la Arrixaca Clinical University Hospital, Murcia, Spain; 3 Alicante Institute for Health and Biomedical Research (ISABIAL), Dr Balmis General University Hospital, Alicante, Spain; 4 Clinical Medicine Department, Miguel Hernández University, San Juan de Alicante, Spain; 5 Rheumatology Department, Dr Balmis General University Hospital, Alicante, Spain; 6 Liver and Digestive Diseases Networking Biomedical Research Centre (CIBERehd), Carlos III Health Institute, Madrid, Spain; 7 Clinical Medicine Department, Institute of Research, Development and Innovation in Health Biotechnology of Elche (IDIBE), Miguel Hernández University, San Juan de Alicante, Spain

**Keywords:** Juvenile idiopathic arthritis, T regulatory cells, Patient-reported outcomes

## Abstract

**Objectives:**

To measure regulatory T cell (Treg) levels in the peripheral blood of children with juvenile idiopathic arthritis (JIA) and analyse the association of this measure with disease activity, quality of life, adjustment of treatment, and hospitalisation.

**Methods:**

We conducted a two-phase study (cross-sectional and prospective), including consecutive children with a JIA diagnosis according to ILAR criteria. Our independent variables were Tregs, Th1, Th2, and cytokines in peripheral blood, and our dependent variables in the cross-sectional phase were arthritis category, JIA activity, and patient-reported outcomes. To test associations, we used Spearman’s correlation coefficient and the Mann-Whitney *U* test. In the prospective phase, we explored the probability of treatment adjustment and hospitalisation for JIA during follow-up according to Tregs levels at baseline, using Cox proportional regression.

**Results:**

Our sample included 87 participants (median age 11 years, 63.2% girls). Tregs were not associated with most variables of interest. However, we found that higher Tregs concentration was associated with lower erythrocyte sedimentation rate (ESR) and better subjective disease status and course, while higher IL-10 and TGF-β levels were associated with lower ESR, less pain, and better subjective disease status We found no association between Tregs and treatment adjustments or hospitalisation.

**Conclusions:**

Higher baseline Treg levels in the peripheral blood of children with JIA may be associated with reduced disease activity and better quality of life, though were not informative on the inflammatory progression on the follow-up.

## Significance and innovations

- Treg levels were inversely associated with lower inflammatory markers (ESR) and better disease status and course as perceived by participants and their parents.
- We found no differences in Treg and cytokine levels according to category of JIA.
- Tregs were not predictive of further treatment optimisation or escalation during prospective follow-up.

## Introduction

Juvenile idiopathic arthritis (JIA) refers to different forms of chronic arthritis of unknown origin with onset before age 16. While rare, JIA is the most common childhood rheumatic disorder. One systematic review estimated global incidence and prevalence of JIA in European children at 8.3 per 100,000 and 32.6 per 100,000, respectively [1]. The 2001 International League of Associations for Rheumatology (ILAR) classification, accepted worldwide [2], includes seven JIA categories. However, some studies suggest it does not fully align with the pathophysiological evidence [3, 4]. Efforts are ongoing to revise ILAR criteria to define more homogeneous clinical groups, incorporating laboratory markers and clinical attributes [5].

Modern treatment strategies have markedly improved the outcomes of chronic pain, disability, and quality of life in children with JIA, but there is still room for improvement [6, 7]. Ten years after disease onset, less than 50% of children with JIA achieve drug-free remission [8], though the numbers vary across studies according to the definition of remission [9–11]. Some studies have found that drug-free remission is more common in oligoarticular JIA, and less common in polyarticular and enthesitis-related arthritis (ERA) categories [12, 13]. Certain clinical variables at diagnosis (such as JIA category, joint counts, and pattern of joint involvement) can help to predict subsequent outcomes (mainly remission, disability, and articular damage), though with only moderate accuracy [14, 15]. Consequently, there is a need to identify more accurate predictors.

Different pathogenic mechanisms involved in JIA cause an imbalance between regulatory and effector cells [16, 17], but these mechanisms are not fully understood. Recent discoveries related to biomarkers, genetics, proteomics, and microbiomics could help clinicians to establish a more personalised prognosis and treatment for individuals with JIA [3, 4, 18–20]. Regarding cellular involvement in the disease, there is consistent evidence of an association between effector T cells and JIA pathophysiology, as exemplified by the role of reactive oligoclonal T cell receptor subsets in pro-inflammatory responses and disease activity [16, 17, 21]. Moreover, there is major interest in evaluating the role of regulatory T cells (Tregs, a subset of T helper (Th) cells) in the pathogenesis of autoimmune disease. Tregs are characterised by CD4, CD25, and CD127 markers and are highly enriched for forkhead box P3 (FOXP3) expression. They prevent allergies, maintain self-tolerance, and control immune reactions [22]. Research has demonstrated the potential role of Tregs in some rheumatic diseases, such as systemic lupus erythematosus (SLE) [23], rheumatoid arthritis [24], autoimmune myositis [25], and systemic sclerosis [26]. In JIA, some studies have assessed plasma and synovial Treg levels, but the combined evidence is insufficient to draw robust conclusions about their implication in the disease owing to small simple size and varied results across different studies [27, 28]. Interestingly, some data suggest T cells can display effector and regulatory function, and this plasticity may play an important role in the pathogenesis of JIA [29, 30]. Evidence on the regulatory mechanism in JIA may help us to better understand the variability among JIA categories and to establish more accurate prognoses.

We aimed to measure Treg levels in the peripheral blood of children with JIA and analyse the association of this measurement with JIA category, disease activity, quality of life, and subsequent outcomes during follow-up.

## Materials and methods

Our study comprised two phases. In the first phase, we performed a cross-sectional assessment in patients with JIA to gauge differences in Tregs and cytokines according to JIA categories and other disease features. In the second phase, we conducted prospective follow-up to evaluate whether Tregs could predict treatment adjustment or hospitalisation. Our study was carried out in two hospitals in south-eastern Spain.

We included consecutive children with a JIA diagnosis according to the ILAR criteria [2] when they attended the rheumatology clinics of participating hospitals during the recruitment period (February 2019 to July 2020). The follow-up period for all participants was until February 2022. Our only exclusion criterion was refusal to participate in the study. The ethics committees of both hospitals approved the study (reference number 2018-7-3-HCUVA for the Clinical Research Ethics Committee of Virgen de la Arrixaca hospital, and reference number 190,626 for the Drug Research Ethics Committee of Alicante Health Department – General Hospital). We obtained written informed consent from participants or their parents in accordance with Spanish legislation. Our study meets the Declaration of Helsinki criteria.

### Baseline visit

#### Primary variables

Our independent variables were the percentage of Treg, Th1, and Th2 populations measured by flow cytometry in peripheral blood. T cell subsets were analysed in fresh samples within 24 h of blood collection and stained as previously described [31]. Briefly, we detected Tregs by performing surface staining with CD3:APC, CD4:FITC, and CD25:PE-Cy7 followed by intracellular staining with FoxP3:PE or its corresponding isotype control. To quantify Th1 and Th2 peripheral responses, we performed surface staining using CD3:APC and CD4:FITC antibodies followed by intracellular staining with T-bet: PE, Gata3:BV421, or their respective isotype controls. We purchased all antibodies from Becton Dickinson, acquired tubes using a FACS Canto II flow cytometer, and analysed data with FlowJo cell analysis software (Becton Dickinson). We described Tregs as CD3^+^CD4^+^CD25^+^FoxP3^+^, Th1 as CD3^+^CD4^+^T-bet^+^, and Th2 as CD3^+^CD4^+^Gata3^+^; and we expressed values for Treg, Th1, and Th2 peripheral responses as the percentage of total CD3^+^CD4^+^ cells (Th cells). Supplementary Fig. 1 shows a representative gating strategy used in this study.

We also measured a panel of pro- and anti-inflammatory cytokines (interferon-gamma (IFN-γ), interleukin (IL)-4, IL-6, IL-10, and transforming growth factor beta (TGF-β)) in serum samples of all participants using enzyme-linked immunosorbent assays (ELISAs). Serum samples for ELISA determinations were obtained after gradient centrifugation of peripheral blood and stored at − 20 °C. We used Quantikine Human ELISA kits from R&D Systems (Minneapolis, MN), following the manufacturer’s instructions for all assays. All samples were tested in triplicate and read in an automated microplate reader. Standard curves were generated for each plate, and the average zero standard optical densities were subtracted from the rest of standards, controls, and samples to obtain a corrected concentration for all cytokines.

#### Secondary variables

Our secondary variables at baseline included clinical and laboratory data, disease activity, and functional status.

For *clinical variables*, we recorded JIA category according to ILAR classification [2]. In view of the clinical heterogeneity in JIA and recent reclassification proposals based on genetic and phenotypic features, we also explored the following groups: systemic JIA, oligoarticular-persistent JIA, oligoarticular-extended JIA, rheumatoid factor (RF)-negative polyarticular JIA, RF-positive polyarticular JIA, ERA, psoriatic JIA, and undifferentiated JIA [3]. Moreover, we subclassified cases according to the age of onset (< six years (early) versus ≥ six years (late)) [5]. Furthermore, we recorded age, sex, current and previous treatments (including intra-articular agents), presence of extra-articular manifestations, and current joint counts. Laboratory variables of interest were the acute-phase reactants C-reactive protein (CRP) and erythrocyte sedimentation rate (ESR), as well as blood counts. To obtain the clinical and laboratory data, we reviewed medical records, conducted face-to-face interviews with participants and parents on the day of enrolment, performed physical examinations, and took blood samples.

To determine *disease activity* and *functional status*, we used Wallace criteria and the Juvenile Arthritis Disease Activity Score (JADAS). The JADAS comprises four variables: physician global assessment, parent/participant global assessment, active joint count (different versions of the instrument test 71, 27, or 10 joints; we used all three versions), and acute-phase reactant (we used the versions that include ESR and CRP [32], and also the three-variable JADAS without an acute-phase reactant [33]).

To record *quality of life*, we used the Juvenile Arthritis Multidimensional Assessment Report (JAMAR), which groups the main patient-reported outcomes (PROs) relevant to JIA and provides clinicians with a complete overview of patients’ status. [34]. This questionnaire includes 15 measures: functional ability on a scale of 0 to 30; pain intensity on a 10-cm visual analogue scale (VAS); quality of life on the Paediatric Rheumatology Quality of Life Scale (PRQL), with a total score ranging from 0 to 30 (0 to 15 for physical health and 0 to 15 for psychosocial health); overall patient wellbeing on a 10-cm VAS; pain or swelling in joints; morning stiffness; extra-articular symptoms; level of disease activity on a 10-cm VAS; disease status (remission, continued activity, or relapse); disease course (much improved, slightly improved, stable, slightly worsened, much worsened) since last visit; drugs; side effects; difficulties with medication; school problems related to JIA; and a question about satisfaction with the disease outcome.

### Follow-up assessment

After the enrolment visit, we reviewed each participant’s electronic case reports over the follow-up period. The *outcome variables of interest* were the occurrence of treatment adjustment or hospitalisation. Treatment optimisation was considered when treating clinical decided any dose reduction (with no specific threshold in mg/kg/day) or suspension of glucocorticoids, disease-modifying drugs, or biological therapies. Treatment intensification was defined as administration of intra-articular injections, any dose increase, or initiation of glucocorticoids, disease modifying drugs, or biological therapy.

We only counted hospitalisations due to disease activity and not due to infectious complications, drug toxicity, or other concurrent complications, according to electronic discharge reports.

The follow-up period was from date of enrolment until the outcome or end of follow-up (28 February 2022).

### Statistical analysis

For quantitative variables, including the independent variable (Tregs, expressed as percentage of total CD3^+^CD4^+^ cells in peripheral blood), we reported medians and interquartile ranges (IQRs) because the Kolmogorov-Smirnov test showed a non-normal distribution. For categorical variables, we reported absolute and relative frequencies. As there are no available reference values for Tregs, we also categorised this variable at its median (below or above 1.67%) and in its terciles (T1: below 1.29%; T2: 1.29–2.18%; T3: above 2.18%) to assess associations.

To study the association of Treg levels with other clinical and laboratory variables, we used Spearman’s rank correlation coefficient for quantitative variables, the Mann-Whitney *U* test for binary variables, and the Kruskal-Wallis *H* test for multicategorical variables. Spearman’s coefficient ranges from − 1 to 1. There are several possible approaches to linking grades with a weak, moderate, or strong relation; for this study we used the following cutoff points [35]: 0.00–0.09, no correlation; 0–10–0.39, weak correlation; 0.40–0.69, moderate correlation; 0.70–0.89, strong correlation; 0.90–1.00, very strong correlation.

In the longitudinal phase of the study, we registered the occurrence of the outcomes of interest (treatment optimisation, treatment escalation, hospitalisation due to disease activity) and calculated their incidence rate. The index date was the date of enrolment in the study, and the final date was when the outcome occurred or the end of follow-up (28 February 2022). We used a Cox regression model to define the associations of the baseline levels of Tregs, other T cells, and cytokines with the different outcomes as hazard ratios (HRs) with their 95% confidence intervals (CIs). Where we found multiple variables with a significant association, we used multivariable adjustment, unless there was a close association with other covariates.

We performed the statistical analyses with SPSS statistical software v25 (IBM, Armonk, NY), defining the level of significance as *P* < 0.05.

## Results

Our study included 87 children. All the children we approached agreed to participate.

### Baseline visit

#### Clinical characteristics

**Fig. 1:**
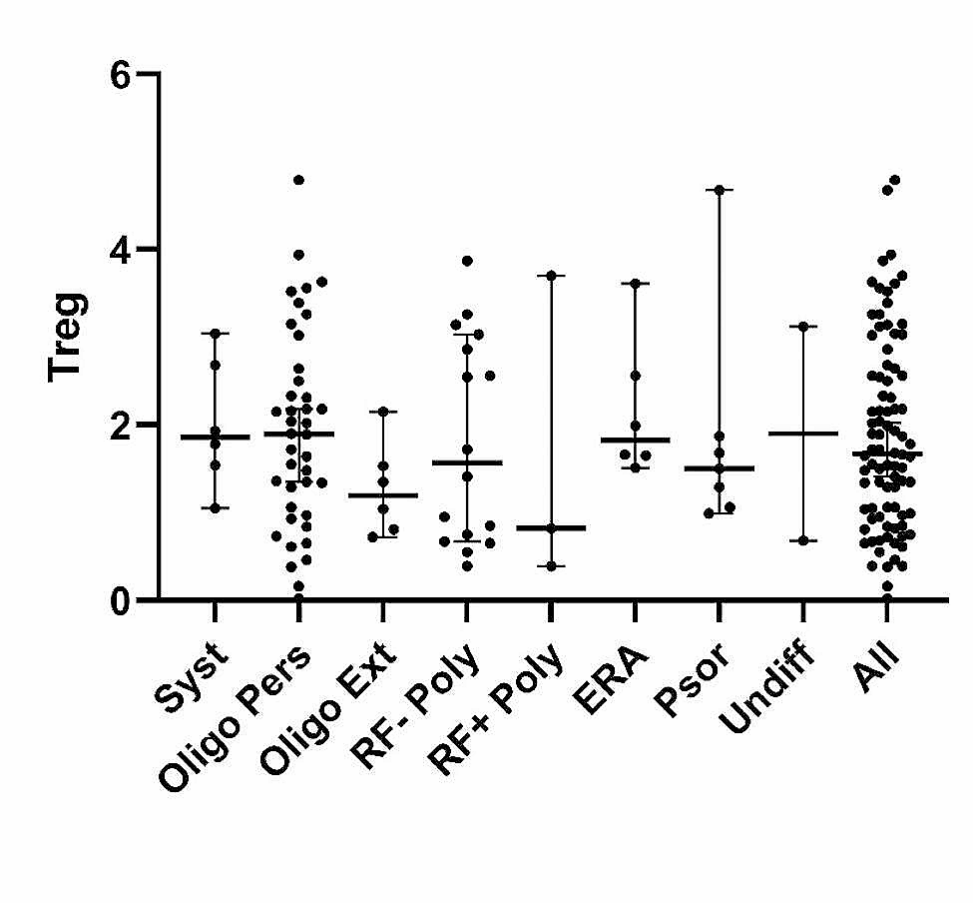
Level of regulatory T cells (as % of total CD3^+^CD4^+^) according to JIA category, represented as mean and 95% confidence interval. ERA: enthesitis-related arthritis; Oligo Ext: oligoarticular-extended arthritis; Oligo Pers: oligoarticular-persistent arthritis; Psor: psoriatic arthritis; RF- Poly: rheumatoid factor-negative polyarticular arthritis; RF + Poly: rheumatoid factor-positive polyarticular arthritis; Syst: systematic arthritis; Undiff: undifferentiated arthritis

Table 1 shows participants’ clinical and laboratory data. The median age was 11 years (IQR 7 to 15), 63.2% were girls, and the most common JIA category was oligoarticular-persistent (44.8%) (Fig. 1). At baseline, 79.3% of participants were in remission according to the Wallace criteria. Participants’ and parents’ perception of disease activity was remission in 62.7% of cases, persistent activity in 20.5%, and relapse in 16.9%. The vast majority of participants were satisfied with disease course (90.4%).

**Table 1:** Demographic and clinical characteristics of the study population (*n* = 87) at baseline

Variables	Data^a^
Demographic characteristics	
Sex (female)	55 (63.2%)
Current age in years, median (IQR)	11 (7; 15)
***JIA characteristics***	
Disease duration at the last visit, median (IQR)	2 (5; 9)
JIA category	
Systemic	6 (6.9%)
Oligoarticular, persistent	39 (44.8%)
Oligoarticular, extended	6 (6.9%)
Polyarticular RF negative	16 (18.4%)
Polyarticular RF positive	3 (3.4%)
Psoriatic	7 (8.0%)
ERA	6 (6.9%)
Undifferentiated	4 (4.6%)
Extra-articular manifestations	24 (27.6%)
Uveitis	10 (11.5%)
Psoriasis	8 (9.2%)
Others^b^	6 (6.8%)
***Treatment***	
No current treatment	29 (33.3%)
Conventional synthetic DMARDs	42 (48.3%)
Biological DMARDs	40 (46.0%)
***Laboratory data***	
ESR (mm/h), median (IQR)^c^	6 (2; 13)
CRP (mg/dL), median (IQR)^d^	0.08 (0.05; 0.22)
***Disease activity state***	
Remission (by Wallace criteria)	69 (79.3%)
Remission (by JADAS27-ESR < 1)^c^	42 (50.6%)
Remission (by JADAS27-CRP < 1)^d^	45 (52.3%)
***Quality of life***	
PRQL – JAMAR, median (IQR)^c^	1 (0; 5)
Physical (JAMAR), median (IQR)^c^	1 (0; 3)
Psychosocial (JAMAR), median (IQR)^c^	0 (0; 2)
Satisfaction with disease outcome (JAMAR)^c^	75 (90.4%)

^a^Data shown as frequencies and percentages, unless specified otherwise

^b^Inflammatory bowel disease and more than one type

^c^This analysis included 83 participants

^d^This analysis included 86 participants

CRP: C-reactive protein; DMARDs: disease modifying anti-rheumatic drugs; ERA: enthesitis related arthritis; ESR: erythrocyte sedimentation rate; IQR: interquartile range; JADAS: Juvenile Arthritis Disease Activity Score; JAMAR: Juvenile Arthritis Multidimensional Assessment Report; JIA: juvenile idiopathic arthritis; PRQL: Pediatric Rheumatology Quality of Life Scale; RF: rheumatoid factor

The median levels of T cells and cytokines were: Tregs, 1.67% (IQR 0.96–2.58); Th1, 1.15% (IQR 0.42–2.07); Th2, 1.20% (IQR 0.74–1.86); IFN-γ, 4.34 pg/mL (IQR 3.23–8.39); IL-6, 13.73 pg/mL (IQR 10.74–17.23); IL-4, 3.66 (IQR 3.20–4.31); IL-10, 7.17 pg/mL (4.19–9.54); TGF-β, 5.95 pg/mL (4.33–7.25).

#### Association between tregs and variables of interest at baseline

Tables 2 and 3 show the associations of out independent variables with quantitative and qualitative dependent variables, respectively. We found no associations between Treg levels and sex, age, or JIA category according to ILAR criteria (Fig. 1); nor was there an association with JIA category according to the five-group reclassification (*p* = 0.733). We found no differences in Treg levels when we compared oligoarticular-persistent versus oligoarticular-extended plus RF-negative polyarticular JIA, versus psoriatic plus enthesitis related arthritis, and oligoarticular extended plus negative RF polyarticular plus psoriatic and enthesitis related arthritis reported no differences among groups (*p* = 0.340, *p* = 0.965, *p* = 0.204 respectively). No other group comparisons were possible because there were very few individuals in some categories. Treg levels were similar in patients with early disease onset and late disease onset (1.68% (IQR 1.05–2.90) versus 1.67% (IQR 0.77–2.50); *p* = 0.561).

**Table 2:** Correlation between levels of peripheral T cells (%) and cytokines (pg/mL) and continuous variables of interest at baseline visits, expressed as Spearman’s rank correlation coefficient with its p value in parenthesis

	Treg	Th1	Th2	IFN-γ	IL-6	IL-4	IL-10	TGF-β
**Age at onset**	−0.033 (0.766)	−0.051 (0.644)	0.155 (0.160)	−0.093 (0.402)	−0.109 (0.325)	−0.070 (0.524)	0.002 (0.982)	0.030 (0.784)
**ESR (mm/hr)**	−**0.247 (0.025)**	0.029 (0.801)	−0.017 (0.884)	0.004 (0.970)	0.111 (0.326)	−0.119 (0.292)	**−0.257 (0.021)**	**−0.267 (0.017)**
**CRP (mg/dL)**	−0.150 (0.172)	−0.159 (0.151)	−0.010 (0.929)	−0.075 (0.500)	−0.066 (0.553)	**−0.247 (0.024)**	−0.147 (0.185)	−0.200 (0.069)
**Active joints**	−0.058 (0.593)	−0.027 (0.808)	−0.146 (0.186)	−0.049 (0.660)	0.009 (0.932)	**−0.302 (0.005)**	−0.078 (0.483)	−0.111 (0.314)
**JADAS27-ESR**	−0.084 (0.456)	−0.063 (0.578)	−0.150 (0.183)	−0.100 (0.379)	−0.033 (0.772)	−0.214 (0.056)	−0.141 (0.213)	−0.211 (0.060)
**JADAS27-CRP**	−0.048 (0.664)	−0.086 (0.439)	−0.148 (0.181)	−0.105 (0.346)	−0.049 (0.660)	−0.159 (0.152)	−0.095 (0.395)	−0.167 (0.132)
**VAS - pain (JAMAR)**	−0.161 (0.149)	−0.142 (0.210)	−0.073 (0.522)	−0.166 (0.142)	−0.126 (0.267)	−0.031 (0.785)	**−0.220 (0.050)**	**−0.327 (0.003)**
**VAS - wellbeing (JAMAR)**	−0.048 (0.667)	−0.056 (0.624)	0.030 (0.789)	−0.072 (0.528)	−0.028 (0.803)	−0.140 (0.215)	−0.121 (0.285)	−0.174 (0.122)
**VAS - activity (JAMAR)**	−0.034 (0.761)	−0.101 (0.374)	−0.151 (0.181)	−0.123 (0.277)	−0.090 (0.427)	−0.123 (0.278)	−0.064 (0.576)	−0.172 (0.127)
**PRQL (JAMAR)**	−0.132 (0.237)	−0.025 (0.825)	−0.019 (0.865)	−0.086 (0.449)	0.002 (0.989)	−0.171 (0.129)	−0.177 (0.087)	**−0.298 (0.007)**
**PhQL (JAMAR)**	−0.141 (0.207)	−0.041 (0.717)	−0.065 (0.570)	−0.111 (0.326)	−0.037 (0.747)	**−0.232 (0.039)**	−0.071 (0.532)	**−0.305 (0.006)**

In bold, statistically significant results

CRP: C-reactive protein; ESR: erythrocyte sedimentation rate; IFN-γ: interferon gamma; IL: interleukin; JADAS: Juvenile Arthritis Disease Activity Score; JAMAR: Juvenile Arthritis Multidimensional Assessment Report; PhQL: Physical Quality of Life; PRQL: Pediatric Rheumatology Quality of Life Scale; TGF-β: transforming growth factor beta; Treg: regulatory T cell; VAS: visual analogue scale

**Table 3:** Association between levels of peripheral T cells (%) and cytokines (pg/mL) and categorical variables of interest at baseline visits

	Treg	Th1	Th2	IFN-γ	IL6	IL4	IL10	TGF-β
Sex								
Female	1.70 (1.00; 2.53)	1.00 (0.41; 1.93)	1.21 (0.74; 1.88)	4.24 (3.35; 8.08)	13.51 (10.63; 17.80)	3.80 (3.14; 4.59)	6.85 (4.09; 9.16)	5.62 (4.23; 6.86)
Male	1.78 (1.05; 3.03)	1.26 (0.46; 2.08)	1.18 (0.73; 1.57)	4.90 (3.10; 8.80)	15.34 (11.00; 16.95)	3.62 (3.24; 4.08)	8.06 (4.48; 9.73)	6.12 (5.07; 7.65)
*p value*	0.197	0.464	0.516	0.974	0.713	0.685	0.155	0.080
**JIA category**								
Systemic	1.85 (1.41; 2.77)	0.65 (0.42; 1.69)	1.30 (0.98; 1.78)	3.9 (3.08; 6.17)	12.44 (10.99; 17.89)	4.59 (3.78; 5.10)	7.85 (5.74; 9.74)	6.19 (5.31; 7.46)
Oligoarticular (persistent)	1.96 (1.23; 2.73)	1.15 (0.41; 1.97)	1.07 (0.73; 1.83)	4.65 (3.15; 8.19)	13.73 (10.66; 17.47)	3.61 (3.20; 4.07)	7.44 (4.81; 9.26)	6.23 (4.19; 7.49)
Oligoarticular (extended)	1.35 (0.92; 1.84)	2.58 (0.29; 2.97)	1.45 (0.83; 3.19)	8.36 (3.60; 9.69)	17.99 (10.79; 18.15)	4.58 (3.58; 6.05)	5.22 (3,91; 7.76)	5.07 (4.29; 5.91)
Polyarticular (RF-negative)	1.72 (0.75; 3.03)	0.61 (0.21; 1.79)	1.49 (0.55; 2.41)	4.00 (3.20; 10.30)	14.28 (10.42; 16.87)	4.00 (3.14; 5.20)	5.48 (3.58; 9.63)	6.05 (3.66; 7.65)
Polyarticular (RF-positive)	0.82 (0.39; NC)	1.1 (0.23; NC)	0.79 (0.66; NC)	3.86 (3.60; NC)	13.29 (10.44; NC)	3.14 (3.00; NC)	3.54 (3.36; NC)	4.33 (2.99; NC)
ERA	1.99 (1.65; 3.08)	2.08 (0.35; 2.42)	1.19 (0.55; 1.70)	8.30 (3.04; 9.07)	16.48 (10.54; 17.23)	3.50 (2.74; 4.70)	8.42 (7.50; 9.86)	6.28 (5.29; 7.96)
Psoriatic	1.50 (1.06; 1.87)	1.20 (0.60; 2.36)	1.28 (0.83; 1.89)	4.04 (3.50; 8.80)	13.55 (12.06; 16.95)	3.60 (3.26; 4.28)	6.66 (5.24; 7.75)	5.59 (5.07; 6.80)
Undifferentiated	1.24 (0.75; 2.71)	0.73 (0.12; 1.87)	0.91 (0.66; 1.30)	3.56 (2.64; 7.01)	11.91 (8.50; 16.18)	3.47 (3.11; 3.78)	5.63 (3.43; 9.87)	5.06 (3.86; 6.16)
*p value*	0.687	0.834	0.887	0.777	0.911	0.332	0.544	0.400
**Intra-articular injections**								
No	1.70 (0.89; 2.81)	0.57 (0.21; 1.17)	1.20 (0.67; 1.63)	3.78 (3.05; 4.33)	12.35 (10.29; 14.62)	3.97 (3.27; 5.10)	7.22 (3.74; 9.63)	5.82 (4.30; 7.24)
Yes	1.72 (1.06; 2.56)	1.38 (0.45; 2.36)	1.18 (0.74; 1.89)	4.90 (3.50; 8.80)	14.29 (10.68; 17.26)	3.62 (3.20; 4.30)	7.16 (4.22; 9.35)	5.91 (4.36; 7.18)
*p value*	0.963	**0.016**	0.671	**0.019**	**0.040**	0.363	0.501	0.757
**Systemic therapy**								
No treatment	1.99 (1.29; 2.86)	0.60 (0.21; 1.88)	1.28 (0.74; 1.90)	4.00 (3.05; 7.75)	12.5 (10.20; 16.80)	3.91 (3.26; 4.40)	8.31 (5.22; 10.04)	6.28 (4.80; 7.63)
csDMARD only	1.65 (0.69; 2.46)	1.21 (0.29; 1.92)	1.13 (0.80; 1.80)	3.91 (3.08; 9.02)	13.80 (10.30; 15.90)	3.67 (3.23; 4.51)	7.05 (3.77; 9.40)	5.51 (3.60; 7.03)
bDMARD only	1.48 (1.05; 2.15)	1.31 (0.47; 3.63)	1.07 (0.65; 2.03)	4.90 (3.21; 13.5)	14.20 (10.6; 18.6)	3.95 (3.50; 5.10)	5.94 (4.48; 7.26)	5.26 (4.29; 6.25)
csDMARD and bDMARD	1.87 (0.93; 3.09)	1.26 (0.57; 2.21)	1.09 (0.74; 1.76)	4.70 (3.98; 8.26)	14.20 (12.20; 18.20)	3.24 (3.02; 3.80)	7.62 (3.92; 9.19)	6.00 (4.35; 7.23)
*p value*	0.711	0.425	0.933	0.464	0.498	0.070	0.481	0.326
**Remission (Wallace criteria)**								
No	1.58 (1.03; 2.39)	1.21 (0.39; 2.33)	1.10 (0.72; 1.40)	4.80 (3.09; 8.32)	14.69 (10.28; 17.06)	3.26 (3.03; 4.08)	6.99 (4.15; 8.53)	5.75 (4.19; 7.00)
Yes	1.78 (1.01; 2.66)	1.05 (0.44; 1.99)	1.22 (0.74; 1.91)	4.20 (3.35; 8.38)	13.58 (10.93; 17.22)	3.80 (3.23; 4.49)	7.26 (4.18; 9.60)	6.05 (4.33; 7.23)
*p value*	0.746	0.944	0.449	0.781	0.939	0.056	0.679	0.520
**Status of disease (JAMAR)**								
Remission	2.09 (1.30; 2.98)	1.26 (0.53; 2.41)	1.32 (0.74; 1.91)	4.55 (3.67; 8.82)	14.06 (11.97; 17.25)	3.80 (3.28; 4.40)	8.04 (5.08; 9.70)	6.30 (4.88; 7.57)
Continued activity	1.04 (0.66; 1.78)	0.88 (0.38; 1.61)	0.79 (0.69; 1.34)	4.28 (2.98; 8.03)	14.28 (10.33; 16.71)	3.60 (3.07; 4.19)	4.22 (3.63; 7.28)	4.33 (3.28; 5.95)
Relapse	1.66 (1.45; 3.05)	1.29 (0.40; 2.74)	1.15 (0.70; 2.14)	5.00 (3.40; 8.58)	14.61 (10.82; 18.07)	3.17 (3.00; 4.75)	7.12 (6.06; 9.64)	5.75 (5.00; 7.62)
*p value*	**0.036** *†*	0.500	0.498	0.393	0.761	0.232	**0.021** *†*	**0.006** *†*
**Course of disease (JAMAR)**								
Much improved	2.15 (1.36; 3.39)	1.47 (0.61; 2.58)	1.09 (0.74; 2.08)	7.75 (3.70; 9.60)	15.40 (12.26; 18.14)	3.80 (3.28; 4.40)	8.54 (5.17; 10.24)	6.80 (5.07; 7.66)
Slightly improved	1.66 (1.64; 3.26)	1.26 (0.36; 2.32)	1.12 (0.58; 2.03)	5.40 (3.08; 8.94)	13.58 (10.62; 16.48)	3.40 (2.80; 5.50)	8.06 (6.68; 9.63)	5.75 (5.24; 8.44)
Stable	1.41 (0.77; 2.17)	0.96 (0.38; 1.42)	1.22 (0.74; 1.74)	4.11 (3.12; 5.30)	13.51 (10.30; 16.14)	3.75 (3.15; 4.23)	5.94 (3.68; 8.51)	5.14 (3.82; 6.21)
Slightly worsened	1.29 (1.01; 1.89)	1.39 (0.48; 3.31)	1.35 (0.75; 2.60)	4.60 (3.39; 10.01)	15.68 (10.61; 18.84)	3.34 (2.95; 5.30)	6.05 (3.67; 7.39)	5.48 (4.17; 6.29)
Much worsened	NC	NC	NC	NC	NC	NC	NC	NC
*p value*	**0.045** *††*	0.276	0.886	0.219	0.525	0.627	0.050	0.068

Data expressed as medians (interquartile range). In bold, statistically significant results. *†* All comparisons within pairs of categories also yielded significant results. *††* In single comparisons, Tregs in the “much improved” category were significantly higher than in “stable” or in “slightly worsened”, while were not different to those that “slightly improved”

bDMARD: biological disease-modifying antirheumatic drug; csDMARD: conventional synthetic disease-modifying antirheumatic drug; ERA: enthesitis related arthritis; IFN-γ: interferon gamma; IL: interleukin; JADAS: Juvenile Arthritis Disease Activity Score; JAMAR: Juvenile Arthritis Multidimensional Assessment Report; NC: not calculated (due to very low numbers in this category); PhQL: Physical Quality of Life; PRQL: Pediatric Rheumatology Quality of Life Scale; RF: rheumatoid factor; TGF-β: transforming growth factor beta; Treg: regulatory T cell; VAS: visual analogue scale

Interestingly, our results suggest that high levels of Tregs are associated with lower ESR levels and better perception of course and status of the disease according to the JAMAR questionnaire; while the anti-inflammatory cytokines IL-10 and TGF-β are associated with lower ESR levels, less pain, and better perception of disease status.

### Follow-up assessment

#### Outcomes during follow-up and Tregs as predictors

Table 4 presents the results at the end of follow-up. There were 28 treatment intensifications and 31 optimisations. The most common category of intensification was initiation of a biological drug (21%), while the most common category of optimisation was suspension of a biological drug (29%). The median length of follow-up was 26 months (IQR 12–32) for optimisation and 26 months (14–31) for intensification. There were no hospitalisations due to disease activity, and we found no association between baseline Tregs and therapy intensification (indicative of disease worsening) or optimisation (indicative of disease remission).

**Table 4:** Association between baseline levels of peripheral T-cells and cytokines and treatment changes during follow-up

	OPTIMISATION		INTENSIFICATION	
	HR (95% CI)	*p* value	HR (95% CI)	*p* value
**Tregs**	0.76 (0.53; 1.11)	0.140	1.03 (0.73; 1.44)	0.882
**Th1 cells**	1.06 (0.91; 1.22)	0.455	1.00 (0.84; 1.19)	0.989
**Th2 cells**	0.74 (0.48; 1.13)	0.162	1.19 (0.95; 1.47)	0.128
**IFN-γ**	1.02 (0.95; 1.09)	0.659	1.00 (0.92; 1.08)	0.997
**IL-6**	1.01 (0.99; 1.03)	0.454	0.99 (0.94; 1.04)	0.687
**IL-4**	0.84 (0.60; 1.19)	0.319	1.08 (0.79; 1.49)	0.621
**IL-10**	0.92 (0.81; 1.05)	0.223	0.99 (0.87; 1.14)	0.928
**TGF-β**	0.79 (0.64; 0.98)	**0.031**	1.03 (0.84; 1.25)	0.812

Optimisation: reduction or cessation of glucocorticoids, disease-modifying drugs, or biological therapy; Intensification: intra-articular injections; increase or initiation of glucocorticoids, disease modifying drugs, or biological therapy

CI: confidence interval; HR: hazard ratio; IFN-γ: interferon gamma; IL: interleukin; TGF-β: transforming growth factor beta; Th: T helper; Tregs: regulatory T cells

## Discussion

Previous studies have examined the role of Tregs in JIA aetiopathogenesis, mainly in oligoarticular and polyarticular categories, focusing on their frequency and ability to control inflammatory response [27, 28, 36–38]. Here, we examined the association between Tregs and relevant variables in clinical practice, such as inflammatory markers, disease activity, and PROs, to establish whether Tregs may be a useful marker in routine care. After analysing Tregs and cytokines in 87 children with all categories of JIA in a real-life setting, we found no associations for most variables of interest. However, Tregs and cytokines related to immune modulation (IL-10 and TGF-β) were inversely associated with ESR, and IL-10 and TGF-β were also inversely associated with pain. Interestingly, higher levels of Tregs, IL-10, and TGF-β were associated with a subjective perception of better disease status, and higher Tregs levels with better disease course. Furthermore, we were able to include a prospective evaluation to analyse the potential predictive role of Tregs for further treatment adjustments, though we found no relation.

Quality of life and other PROs are important tools in JIA. They reflect patients’ and parents’ perception of the disease and treatments, helping clinicians to make treatment decisions and improve therapeutic compliance [39, 40]. In this sense, one novel finding was the link between subjective perception of disease activity by participants or their parents (e.g., pain, physical quality of life, disease status, disease course) and levels of IL-10 and TGF-β. This suggests that an increased Treg population is associated with upregulated TGF-β and IL-10 production in peripheral blood, likely leading to clinical wellbeing in children with JIA. Conversely, Th2 response (IL-4 levels) was inversely associated with CRP, active joints at baseline, and physical function measured by the JAMAR questionnaire. As Th2 response counteracts inflammation driven by Th1, our findings could reveal the anti-inflammatory pathway for children with JIA in remission [41]. Further research is needed to clarify these results.

Our study included a long follow-up of participants’ disease activity outcomes, assessed as subsequent hospitalisations and treatment adjustment. To the best of our knowledge, there are no similar analyses of Tregs in the JIA literature. No hospitalisation occurred in the follow-up period, and changes in treatment (optimisation and intensification) were unrelated to Treg levels. However, higher TGF-β concentration was associated with lower rates of optimisation therapy. We consider this a meaningful result because TGF-β promotes stability and development of Tregs [42]. The attending physician did not have access to participants’ Treg and cytokine levels, so decisions to optimise or intensify treatment were made on clinical grounds. A possible explanation for the lack of association between Tregs and treatment adjustment is that Tregs were measured at a single time point. Future studies should assess whether repeated measures could better reflect the immune system status and predict outcomes.

One strength of our study was the detailed clinical profiling of participants. We recorded variables related to disease onset, baseline situation, and follow-up. Participants shared their insights of the disease through structured and validated questionnaires. However, we must acknowledge some limitations. We were unable to include synovial fluid analysis and had to use peripheral blood results as a surrogate marker of the joint process. Most participants were in remission with ongoing treatment when they were included the study, which hampered adequate differentiation between disease activity or remission, and may have masked potential influences of therapies. Recruitment of treatment-naïve patients with recent disease onset would be of utmost interest. Nevertheless, we found relevant associations related to ESR levels and participant-reported outcomes. Our sample only included children with JIA, and no control groups. In our study, percentage of Tregs in peripheral blood (1.68%) was lower than previously reported in healthy people (5–10%) [43], in line with previous research [27].

Despite cumulative data suggesting a key role of Tregs as immune modulators in several inflammatory conditions (including JIA), we were unable to confirm this hypothesis, though we found weak associations with ESR levels and participant-reported disease status. Moreover, peripheral Tregs were not predictors of subsequent treatment adjustment (neither escalation nor optimisation). Our findings do not support using peripheral Treg levels as an indicator of disease activity in JIA in clinical practice. Future research should focus on the local function of Tregs (in synovial tissue).

## Conclusion

According to our data, higher Treg levels in the peripheral blood of children with JIA may be associated with reduced disease activity and better quality of life, but they were not predictive of inflammatory course during follow-up. However, with future advancements in artificial intelligence and big data, Tregs may become a valuable measure for such purposes.

## Data Availability

The datasets used and/or analysed during the current study are available from the corresponding author on reasonable request.

## Abbreviations

CRP: C-reactive protein
CI: Confidence interval
ELISA: Enzyme-linked immunosorbent assays
ERA: Enthesitis-related arthritis
ESR: Erythrocyte sedimentation rate
FOXP3: Forkhead box P3
HRs: Hazard ratios
IFN: γ-Interferon-gamma
IL: Interleukin
ILAR: International League Associations of Rheumatology
IQR: Interquartile range
JADAS: Juvenile Arthritis Disease Activity Score
JAMAR: Juvenile Arthritis Multidimensional Assessment Report
JIA: Juvenile idiopathic arthritis
PROs: Patient-reported outcomes
PRQL: Quality of life on the Paediatric Rheumatology Quality of Life Scale
RF: Rheumatoid factor
TGF: β-Transforming grow factor beta
Th: T helper cells
Tregs: Regulatory T cells
VAS: Visual Analogue Scale
